# Increased muscle tone and contracture late after ischemic stroke

**DOI:** 10.1002/brb3.1509

**Published:** 2020-01-01

**Authors:** Carina U. Persson, Lukas Holmegaard, Petra Redfors, Christina Jern, Christian Blomstrand, Katarina Jood

**Affiliations:** ^1^ Institute of Neuroscience and Physiology Department of Clinical Neuroscience, Rehabilitation Medicine Sahlgrenska Academy at the University of Gothenburg Gothenburg Sweden; ^2^ Region Västra Götaland Department of Physiotherapy Sahlgrenska University Hospital/Östra Gothenburg Sweden; ^3^ Department of Clinical Neuroscience Institute of Neuroscience and Physiology Sahlgrenska Academy at the University of Gothenburg Gothenburg Sweden; ^4^ Region Västra Götaland Department of Neurology Sahlgrenska University Hospital Gothenburg Sweden; ^5^ Department of Clinical Pathology and Genetics Institute of Biomedicine Sahlgrenska Academy at the University of Gothenburg Gothenburg Sweden; ^6^ Stroke Centre West Sahlgrenska Academy at the University of Gothenburg Gothenburg Sweden; ^7^ Region Västra Götaland Department of Clinical Genetics and Genomics Sahlgrenska University Hospital Gothenburg Sweden

**Keywords:** muscle spasticity, rehabilitation, stroke

## Abstract

**Background:**

Systematic studies on increased muscle tone and spasticity late after ischemic stroke, without any selection, are limited. Therefore, we aimed to determine the prevalence of increased muscle tone, classical spasticity and contracture and predictors of increased muscle tone seven years after stroke.

**Methods:**

Consecutive patients with acute ischemic stroke <70 years of age (*n* = 411) were recruited to the Sahlgrenska Academy Study on Ischemic Stroke. Symptoms at index stroke were assessed using the Scandinavian Stroke Scale. Seven years after stroke, survivors (*n* = 358) were invited for follow‐up assessments, of whom 292 agreed to participate and 288 contributed data. Muscle tone according to the Modified Ashworth scale, classical spasticity, and contracture was assessed by a neurologist. The associations between increased muscle tone and characteristics at index stroke and recurrent strokes during follow‐up were investigated using logistic regression analysis.

**Results:**

Increased muscle tone was recognized in 99 participants (34%): 94 (33%) in the upper limbs, and 72 (25%) in the lower limbs. Classical spasticity was found in 51 participants (18%) and contracture in 26 (9%). Age (odds ratio [OR] 1.03 [95% confidence interval [CI] 1.00–1.06]), arm paresis (OR 1.76 [95% CI 1.40–2.2]), aphasia (OR 1.68 [95% CI 1.12–2.51]), and facial palsy (OR 2.12 [95% CI 1.10–4.07]) were independent predictors of increased muscle tone.

**Conclusions:**

One‐third of patients with ischemic stroke before 70 years of age showed increased muscle tone at 7‐year follow‐up. Half of them also had classical spasticity. Age, arm paresis, aphasia, and facial palsy at index stroke were predictors of increased muscle tone poststroke.

## Graphical abstract

One‐third of patients with ischaemic stroke before 70 years of age showed increased muscle tone at 7‐year follow‐up. Half of them also had classical spasticity, and almost every tenth had one or more contractures. Increased muscle tone was predicted by age, arm paresis, aphasia, and facial palsy at index stroke.

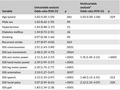

## 1 INTRODUCTION

After stroke, increased muscle tone and spasticity may have a negative impact on health‐related quality of life (Gillard et al., 2015), motor control recovery (Singer, Nishihara, & Mochizuki, 2016), muscle architecture (Dias et al., 2016), and daily activities (Duncan et al., 1997). Increased muscle tone is also associated with fourfold higher direct costs related to hospitalization, municipality services, primary care, and medication (Lundstrom, Smits, Borg, & Terent, 2010). The prevalence of increased muscle tone during the first 12 months after stroke varies between 4% and 46% (Lundstrom, Smits, Borg, et al., 2010; Lundstrom, Terent, & Borg, 2008; Opheim, Danielsson, Alt Murphy, Persson, & Sunnerhagen, 2014; Shin et al., 2018; Sommerfeld, Eek, Svensson, Holmqvist, & von Arbin, 2004; Urban et al., 2010; Watkins et al., 2002), probably because of differences in the case‐mix and study design. Studies beyond the first year are scarce. At 18 months after ischemic or hemorrhagic stroke, increased muscle tone was found in 20% of 66 individuals (Welmer, von Arbin, Widen Holmqvist, & Sommerfeld, 2006). However, less is known about the extent to which increased muscle tone affects long‐term stroke survivors.

Most studies investigating increased muscle tone after stroke (Lundstrom, Smits, Borg, et al., 2010; Lundstrom et al., 2008; Opheim et al., 2014; Shin et al., 2018; Sommerfeld et al., 2004; Urban et al., 2010; Watkins et al., 2002) assessed muscle tone using the Modified Ashworth scale (Bohannon & Smith, 1987; Peacock & Staudt, 1991). The Modified Ashworth scale quantifies the resistance that is felt when muscles are passively stretched, but it does not necessarily indicate whether there is classic spasticity or another type of increased muscle tone. According to Lance, spasticity is defined as “a motor disorder characterized by a velocity‐dependent increase in tonic stretch reflexes (muscle tone) with exaggerated tendon jerks, resulting from hyperexitability of the stretch reflex, as one component of the upper motor neuron syndrome”(Lance, 1980). Thus, the reported prevalence of increased muscle tone after stroke may not directly be translated into the prevalence of spasticity in the classical meaning. The distinction may be of importance when adults with limb spasticity are considered for focal pharmacotherapy drugs (Simpson et al., 2016).

The early identification of those who are at risk of developing increased muscle tone late after stroke may offer possibilities for the early treatment and prevention of negative effects on recovery. Previous studies have consistently shown that initial impairments in sensorimotor function are associated with the risk of developing increased muscle tone after stroke (Lundstrom, Smits, Terent, & Borg, 2010; Opheim, Danielsson, Alt Murphy, Persson, & Sunnerhagen, 2015; Urban et al., 2010). The role of age is less clear (Lundstrom et al., 2008; Shin et al., 2018). The present study aimed to investigate the long‐term prevalence of increased muscle tone, classical spasticity, and contracture and to identify the predictors of increased muscle tone seven years after stroke.

## 2 METHODS

### 2.1 Population

The Sahlgrenska Academy Study on Ischaemic Stroke is a prospective and longitudinal study (Jood, Ladenvall, Rosengren, Blomstrand, & Jern, 2005), in which 600 consecutive patients suffering a first‐ever or a recurrent acute ischemic stroke at the age of 18–69 years were recruited at four stroke units in Western Sweden between August 1998 and December 2003. The current study includes the 411 participants who were recruited at the stroke unit at Sahlgrenska University Hospital at Sahlgrenska. Seven years poststroke, those who were still alive (*n* = 358) were invited for follow‐up assessments. The procedures followed were in concordance with the institutional guidelines. All patients gave their written informed consent at baseline. For those patients who were unable to communicate, the next of kin consented. Surviving participants declining participation in assessments at the seven‐year follow‐up did not withdraw their consent for data collected at baseline. The Regional Ethical Review Board in Gothenburg, Sweden, approved this study (REC number: 413–04).

### 2.2 Assessments at index stroke

At index stroke, all patients were examined with brain computed tomography and/or magnetic resonance imaging. Stroke severity was assessed during the first week after admission to the stroke unit using the Scandinavian Stroke Scale (Barber, Fail, Shields, Stott, & Langhorne, 2004). Scandinavian Stroke Scale is an ordinal scale that includes nine items representing consciousness; eye movements; arm, hand and leg motor function; orientation; speech; facial palsy; and gait, and each item has 2–5 response categories. Scandinavian Stroke Scale gives a total score between 0 and 58. Higher scores indicate better neurological function. In all patients, the lowest Scandinavian Stroke Scale score during the first week was registered.

### 2.3 Assessments seven year after index stroke

Seven years after index stroke, neurological deficits were assessed according to the National Institute of Health Stroke Scale (NIHSS) (Brott et al., 1989), which was part of the clinical practice at that time, instead of using the Scandinavian Stroke Scale (Barber et al., 2004). A trained study neurologist conducted passive movements of seven muscle functions in the upper extremities and legs, taking into account hyperreflexia and the velocity‐dependent nature of spasticity. Increased muscle tone was defined as a Modified Ashworth scale score ≥2 in any of the assessed muscle groups. The Modified Ashworth scale is an ordinal scale with five response categories where 0 represents hypotonic, 1 normal, 2 mild, 3 moderate, 4 severe, and 5 extreme muscle tone. During this examination, the neurologist also assessed both spasticity in the classical sense referring to velocity‐dependent increase in tonic stretch reflexes (muscle tone) with exaggerated tendon jerks, corresponding to the Tardieu scale grade 2–4 (Haugh, Pandyan, & Johnson, 2006), and contractures referring to a restricted range of passive joint motion The participants were asked whether they had any ongoing therapy for spasticity. Information about recurrent strokes during follow‐up was obtained from the Swedish National Hospital Discharge Registry and medical records as described (Redfors et al., 2012).

### 2.4 Statistical methods

The data were analyzed using SAS Software version 9.4 (SAS Institute Inc). Ordinal data are presented as medians, min‐max, and interquartile ranges. Seven‐year NIHSS scores were converted into Scandinavian Stroke Scale scores using the 90‐day algorithm: Scandinavian Stroke Scale = 56.68–2.20 × NIHSS (Gray, Ali, Lyden, & Bath, 2009). Univariable and multivariable logistic regressions were performed to assess the impact of baseline variables and recurrent strokes on increased muscle tone seven years poststroke. The dependent variable was increased muscle tone (i.e., a Modified Ashworth scale score ≥2 in any extremity). The covariates were age, sex, vascular risk factors, smoking, recurrent stroke during follow‐up, and neurological deficits expressed by different items of the Scandinavian Stroke Scale at index stroke. The items were handled as ordinal scales in the logistic regression, with odds ratios expressing the effect for one‐unit increase for each item. The multivariable model was obtained from a stepwise forward logistic regression that included variables significant at the 0.10 level from univariable models. The goodness‐of‐fit for the multivariable logistic model was tested by the Hosmer and Lemeshow test. Descriptively, odds ratios with 95% confidence intervals, p‐values, and area under the level of statistical significance were set at *p < *.05 using a 2‐tailed test.

## 3 RESULTS

**Figure 1:**
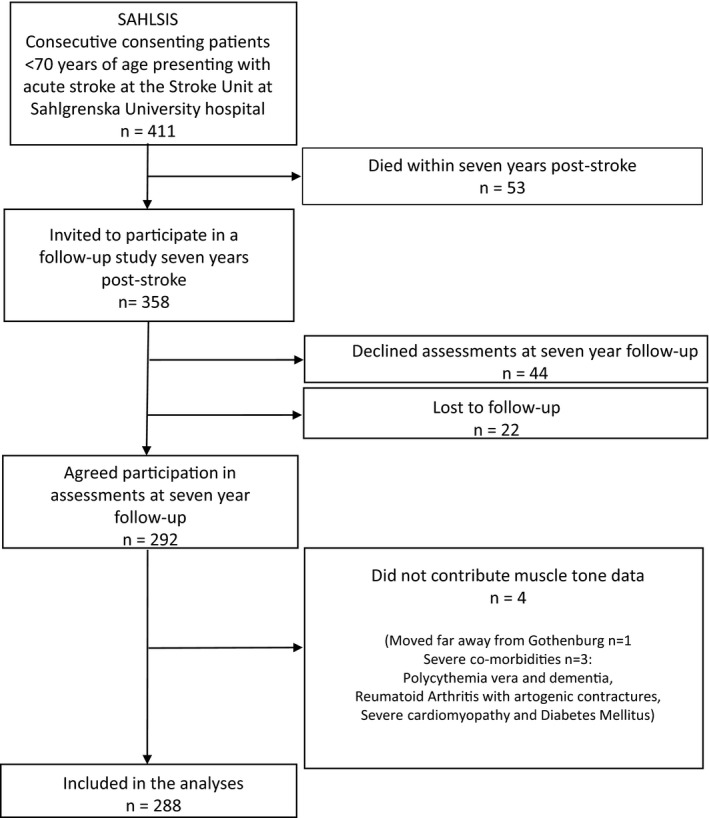
Study flow chart

Of the included 411 patients, 13% had died 7 years after stroke onset. Of the 358 participants who were still alive at the time of follow‐up, 292 (82%) agreed to participate. Forty‐eight of the follow‐ups were home visits. Muscle tone data were collected from 288 participants (Figure 1).

Table 1 summarizes the baseline characteristics at index stroke for those participating and not participating in the follow‐up assessments 7 years after inclusion to the study. Those declining/nonresponders or lost to follow‐up at 7 years poststroke were slightly younger, had somewhat more severe neurological deficits at acute stroke, and were more often smokers. Table 2 shows the Scandinavian Stroke Scale subscores at index stroke for 287 of the participants. At the follow‐up 7 years after the index stroke, the mean and median Scandinavian Stroke Scale scores at follow‐up were 52.0 (8.9 *SD*) and 56.7 (52.3–56.7 IQR), respectively. This corresponds to scores 2.1 (4.1 *SD*) and 0.0 (0.0–2.0 IQR) for the National Institutes of Health Stroke Scale (Gray et al., 2009). At the follow‐up, 35 participants (12%) had experienced a recurrent stroke compared with 6 (9%) among those who did not participate in the follow‐up.

**Table 1:** Baseline characteristics at index stroke

Variable	Participants *N* = 288	Declined to participate in assessment at follow‐up *N* = 66
Age (years) Median (Min‐Max) (IQR)	56.7 (18.8–69.8) (49.8–62.0)	54.3 (18.8–69.1) (44.7–61.2)
Stroke localization		
Right hemisphere, *n* (%)	102 (35.4)	20 (30.3)
Left hemisphere, *n* (%)	128 (44.4)	33 (50.0)
Brainstem, cerebellum, *n* (%)	51 (17.7)	10 (15.2)
More than one location, *n* (%)	7 (2.4)	3 (4.5)
Female Sex, *n* (%)	109 (37.8)	23 (34.8)
Hypertension, *n* (%)	157 (54.5)	32 (48.5)
Diabetes mellitus, *n* (%)	50 (17.4)	10 (15.2)
Current smoker, *n* (%)	101 (35.1)	34 (51.5)
Scandinavian Stroke Scale		
Median score (Min‐Max) (IQR)	54.0 (2.0–58.0) (46.0–57.0)	52.0 (4.0–58.0) (34.0–56.0)

Abbreviations: IQR, interquartile range; *SD*, standard deviation.

**Table 2:** Scandinavian Stroke Scale subscores at index stroke (*N* = 287)

Variable	Response category	*N*	(%)
Consciousness	Fully conscious	263	(91.6)
	Somnolent, can be awakened to fully conscious	18	(6.3)
	Reacts to verbal command, not fully conscious	5	(1.8)
	Reacts to pain only	1	(0.3)
Eye movements	No gaze palsy	262	(91.3)
	Gaze palsy present	18	(6.3)
	Conjugate eye deviation	7	(2.4)
Arm, motor power	Raises hand with normal strength	153	(53.4)
	Raises hand with reduced strength	61	(21.4)
	Raises hand with flexion in elbow	21	(7.3)
	Can move, but not against gravity	5	(1.4)
	Paresis	47	(16.5)
Hand, motor power	Normal strength	143	(49.8)
	Reduced strength in full range	80	(27.9)
	Some movement, fingertips do not reach palm	19	(6.6)
	Paresis	45	(15.7)
Leg, motor power	Normal strength	165	(57.5)
	Raises straight leg with reduced strength	63	(22.0)
	Raises leg with flexion of knee	15	(5.2)
	Can move, but not against gravity	19	(6.6)
	Paresis	25	(8.7)
Orientation	Correct for time, place and person	271	(94.4)
	Two of these correct	4	(1.4)
	One of these correct	3	(1.1)
	Completely disoriented	9	(3.1)
Speech	No aphasia	225	(78.4)
	Limited vocabulary or incoherent speech	36	(12.5)
	More than yes/no, but no longer sentences	8	(2.8)
	Only yes/no or less	18	(6.3)
Facial palsy	None/dubious	187	(65.2)
	Present	100	(34.8)
Gait	Walks 5 m without aids	184	(64.1)
	Walks with aids	18	(6.3)
	Walks with help of another person	18	(6.3)
	Sits without support	26	(9.0)
	Bedridden wheelchair	41	(14.3)

Tables 3 and 4 illustrate the response categories of the Modified Ashworth scale seven years after the index stroke. Increased muscle tone (a Modified Ashworth scale score ≥2), regardless of location, was found in 99 participants (34%); of these 94 (95%) had increased muscle tone in the upper extremities, and 72 (73%) in the legs. For the majority, the increased muscle tone was mild or moderate in both the upper and lower extremities. Increased muscle tone was most prevalent in the elbow flexors (upper extremities) and the knee flexors (lower extremities). The finger flexors in the upper extremities and the plantar flexors in the lower extremities had the highest proportion of severe and extreme muscle tone. Of the 99 participants with increased muscle tone, eight reported that they had been treated with local botulinum toxin injections. One used oral baclofen, seven had orthosis, and 32 reported that they had ongoing physiotherapy. Of the 189 without any increased muscle tone, none had botulinum toxin or baclofen, one had orthosis, and three had ongoing physiotherapy.

**Table 3:** Modified Ashworth scale score for upper extremities 7 years after ischemic stroke (*N* = 288)

Muscles	Muscle tone	*N*	(%)
Shoulder adductors, *N* = 288	Normal	238	(82.6)
	Mild	24	(8.3)
	Moderate	18	(6.3)
	Severe	6	(2.1)
	Extreme	2	(0.7)
Shoulder inward rotators, *N* = 288	Normal	229	(79.5)
	Mild	27	(9.4)
	Moderate	20	(7.0)
	Severe	9	(3.1)
	Extreme	3	(1.0)
Elbow flexors, *N* = 288	Normal	206	(71.5)
	Mild	39	(13.5)
	Moderate	22	(7.6)
	Severe	15	(5.3)
	Extreme	6	(2.1)
Forearm pronators, *N* = 288	Normal	228	(79.2)
	Mild	23	(8.0)
	Moderate	19	(6.6)
	Severe	15	(5.2)
	Extreme	3	(1.0)
Wrist flexors, *N* = 288	Hypotonic	1	(0.3)
	Normal	222	(77.1)
	Mild	26	(9.0)
	Moderate	18	(6.3)
	Severe	15	(5.2)
	Extreme	6	(2.1)
Finger flexors, *N* = 288	Hypotonic	1	(0.3)
	Normal	222	(77.1)
	Mild	24	(8.3)
	Moderate	15	(5.2)
	Severe	20	(7.0)
	Extreme	6	(2.1)
Thumb adductors, *N* = 288	Hypotonic	1	(0.3)
	Normal	226	(78.5)
	Mild	26	(9.0)
	Moderate	18	(6.3)
	Severe	11	(3.8)
	Extreme	6	(2.1)

**Table 4:** Modified Ashworth scale score for lower extremities seven years after ischemic stroke (*N* = 287)

Muscles	Muscle tone	*N*	(%)
Hip adductors, *N* = 287	Normal	244	(85.0)
	Mild	23	(8.0)
	Moderate	14	(4.9)
	Severe	6	(2.1)
Hip flexors, *N* = 287	Normal	244	(85.0)
	Mild	27	(9.4)
	Moderate	11	(3.8)
	Severe	4	(1.4)
	Extreme	1	(0.4)
Knee flexors, *N* = 286	Normal	228	(79.7)
	Mild	27	(9.4)
	Moderate	23	(8.1)
	Severe	8	(2.8)
Knee extensors, *N* = 286	Normal	231	(80.8)
	Mild	29	(10.1)
	Moderate	20	(7.0)
	Severe	6	(2.1)
Ankle flexors, *N* = 286	Normal	226	(79.0)
	Mild	23	(8.0)
	Moderate	16	(5.6)
	Severe	14	(4.9)
	Extreme	7	(2.5)
Plantar flexors, *N* = 286	Normal	228	(79.7)
	Mild	20	(7.0)
	Moderate	15	(5.3)
	Severe	17	(5.9)
	Extreme	6	(2.1)
Toe flexors, *N* = 286	Normal	230	(80.4)
	Mild	23	(8.0)
	Moderate	18	(6.3)
	Severe	10	(3.5)
	Extreme	5	(1.8)

Classical spasticity (Lance, 1980), regardless of localization, was recognized in 51 participants (18%), all of whom also had a Modified Ashworth scale score ≥2. Of these, 46 participants (90%) had spasticity in the upper extremities, 34 participants (67%) in the lower extremities, and 29 participants (57%) in both the upper and lower extremities. Among those with spasticity, six participants reported that they had been treated with botulinum toxin, and one had received baclofen; four had orthosis, and 19 had ongoing physiotherapy. Twenty‐six participants had contracture; among those, all but one also had increased muscle tone. Of those with contracture, 21 participants had contracture in the upper extremities, 17 in the lower limbs, and 11 in both.

Table 5 shows the results of univariable and multivariable stepwise logistic regression analysis for predictors of increased muscle tone seven years poststroke. In the univariable analysis, consciousness, eye movements, arm, hand and leg motor power, orientation, aphasia, facial palsy and gait were all statistically significant predictors, but there was no statistically significant association with age, sex, hypertension, diabetes, smoking, or recurrent stroke. In the multivariable analysis, higher age, arm paresis, aphasia, and facial palsy remained statistically significant predictors of increased muscle tone.

**Table 5:** Univariable and multivariable logistic regression showing the associations between baseline clinical variables, including the Scandinavian Stroke Scale (SSS) subscores at index stroke, as well as recurrent stroke and increased muscle tone seven years poststroke (*N* = 288)

Variable	Univariable analysis Odds ratio (95% CI)	*p*	Multivariable analysis^a^ Odds ratio (95% CI)	*p*
Age (years)	1.02 (1.00–1.05)	.061	1.03 (1.00–1.06)	.029
Male sex	1.03 (0.62–1.70)	.90		
Hypertension	1.44 (0.88–2.37)	.15		
Diabetes mellitus	1.34 (0.72–2.51)	.36		
Smoking	0.97 (0.58–1.66)	.90		
Recurrent stroke	1.97 (0.97–4.02)	.063		
SSS consciousness	2.91 (1.43–5.89)	.0031		
SSS eye movements	2.48 (1.29–4.75)	.0064		
SSS arm motor power	2.11 (1.63–2.57)	<.0001	1.76 (1.40–2.21)	<.0001
SSS hand motor power	2.59 (1.99–3.37)	<.0001		
SSS leg motor power	2.20 (1.75–2.77)	<.0001		
SSS orientation	2.10 (1.27–3.47)	.0037		
SSS speech	2.13 (1.53–2.97)	<.0001	1.68 (1.12–2.51)	.012
SSS facial palsy	5.07 (2.99–8.61)	<.0001	2.12 (1.10–4.07)	.024
SSS gait	1.83 (1.54–2.18)	<.0001		

Note

Increased muscle tone was defined as a Modified Ashworth scale score of ≥2 in any limb. SSS indicates Scandinavian Stroke Scale. For the Scandinavian Stroke Scale items, the odds ratio for a one‐unit increase of the score is given.

a The final multivariable model was obtained from a stepwise logistic regression including variables significant at the 0.10 level from univariable models, and it included age and the items arm motor power, speech and facial palsy from the Scandinavian Stroke Scale. The Hosmer and Lemeshow goodness‐of‐fit had a *p*‐value of .27.

## 4 DISCUSSION

To our knowledge, this is the first study on ischemic stroke in which the prevalence of increased muscle tone and the number of patients meeting the classical spasticity criteria were investigated several years after acute stroke. Seven years after stroke onset, one‐third had increased muscle tone; for the majority, the muscle tone was mildly or moderately increased. Increased muscle tone was predicted by higher age, arm paresis, aphasia, and facial palsy at index stroke. Further, half of those with increased muscle tone displayed classical spasticity, and almost one in ten participants had contractures.

The observed prevalences of both increased muscle tone and spasticity are within the range of the previously reported prevalence of increased muscle tone, as assessed by the Modified Ashworth scale, within the first 18 months after stroke onset (Lundstrom et al., 2008; Opheim et al., 2014; Sommerfeld et al., 2004; Urban et al., 2010; Welmer et al., 2006). As expected and in line with previous research (Picelli et al., 2014; Ryu, Lee, Lee, & Chun, 2010; Urban et al., 2010), motor impairment in the early phase of stroke predicts increased muscle tone at later stages. To our knowledge, aphasia has not been reported as a predictor for increased muscle tone. However, this finding is plausible because such an impairment can indicate larger cortical or subcortical damage. Our study lends further support to the literature on an association between increasing age and risk for increasing muscle tone. In contrast to some previous studies (Lundstrom et al., 2008; Urban et al., 2010; Watkins et al., 2002), sex did not predict the prevalence of increased muscle tone.

The clear difference in the prevalence of increased muscle tone based on the Modified Ashworth scale and classical spasticity indicates that the term spasticity, based on the Modified Ashworth scale data alone, may confuse researchers and clinicians. Thus, further research concerning different types of increased muscle tone in relation to different treatments and outcomes is warranted.

The finding that almost one out of every ten patients had one or more contractures is noteworthy. Our results may indicate suboptimal treatment, related to use, but also to intensity, duration, and frequency, of interventions such as physiotherapy, surgery, and pharmacological treatments. The explanations may include lack of knowledge and resources, limited periods of rehabilitation, and/or ambiguity regarding responsibility for the provision of care in a long‐term perspective (hospital, municipality, and primary care). Thus, our results suggest a need for increased awareness to identify increased muscle tone both in the short‐ and long‐term perspectives after stroke.

The strength of the current study includes the consecutive recruitment and the large and relatively young sample, the long follow‐up time, and that stroke neurologists evaluated classical spasticity and contracture and assessed muscle tone by the Modified Ashworth scale. However, the results must be interpreted within the context of some limitations. The results may not be generalized to hemorrhagic stroke or to older stroke survivors. On the other hand, data on long‐term outcomes are of particular importance for younger stroke survivors, who generally have a longer life expectancy. Although the participation rate was relatively high at the follow‐up (82%), a potential selection bias exists in those who declined participation/did not respond to study invitation or were lost to follow‐up had somewhat more severe strokes according to the Scandinavian Stroke Scale scores at admission. Thus, if anything, this bias is expected to underestimate the frequency of increased muscle tone. As the Modified Ashworth scale is a standard method to screen for increased muscle tone after stroke, we used this scale for the analysis of association between muscle tone at follow‐up and baseline variables. However, as this scale does not take into account the angle of muscle contraction or different speeds (Patrick & Ada, 2006), it must be noted that this is an analysis of increased muscle tone in a broader sense and not specifically classical spasticity. Moreover, we did not use a validated instrument for the assessment of classical spasticity and contracture. However, the assessments were made by two study neurologists specifically trained for grading spasticity and contracture. Another study limitation is that the Scandinavian Stroke Scale was used at baseline in the acute period, while the National Institutes of Health Stroke Scale was used at the follow‐up. However, these instruments show good agreement, and accordingly, an algorithm can be used to convert scores (Gray et al., 2009). Finally, the design did not include repeated assessments during follow‐up. Thus, we cannot draw conclusions about the time course or effects of earlier or ongoing treatments from baseline to follow‐up.

## 5 CONCLUSION

One‐third of patients with ischemic stroke before 70 years of age showed increased muscle tone at seven‐year follow‐up. Half of them also had classical spasticity, and almost every tenth had one or more contractures. Increased muscle tone was predicted by age, arm paresis, aphasia, and facial palsy at index stroke.

## CONFLICT OF INTEREST

The authors declared no potential conflicts of interest with respect to the research, authorship, and/or publication of this article.

## Data Availability

The data that support the findings of this study are available from the corresponding author upon reasonable request.
